# The L444P *Gba1* mutation enhances alpha-synuclein induced loss of nigral dopaminergic neurons in mice

**DOI:** 10.1093/brain/awx221

**Published:** 2017-09-06

**Authors:** Anna Migdalska-Richards, Michal Wegrzynowicz, Raffaella Rusconi, Giulio Deangeli, Donato A Di Monte, Maria G Spillantini, Anthony H V Schapira

**Affiliations:** 1 Department of Clinical Neurosciences, Institute of Neurology, University College London, London NW3 2PF, UK; 2 Department of Clinical Neurosciences, Clifford Allbutt Building, University of Cambridge, Cambridge, CB2 0AH, UK; 3 German Centre for Neurodegenerative Diseases (DZNE), Sigmund-Freud-Strasse 27, 53127 Bonn, Germany; 4 Scuola Superiore Sant’Anna, Piazza Martiri della Libertà 33, 56127 Pisa, Italy; 5 University of Pisa, Lungarno Antonio Pacinotti 43, 56126 Pisa, Italy

**Keywords:** glucocerebrosidase, *GBA1*, alpha-synuclein, Parkinson’s disease, neurodegeneration

## Abstract

Mutations in glucocerebrosidase 1 (*GBA1*) represent the most prevalent risk factor for Parkinson’s disease. The molecular mechanisms underlying the link between *GBA1* mutations and Parkinson’s disease are incompletely understood. We analysed two aged (24-month-old) *Gba1* mouse models, one carrying a knock-out mutation and the other a L444P knock-in mutation. A significant reduction of glucocerebrosidase activity was associated with increased total alpha-synuclein accumulation in both these models. *Gba1* mutations alone did not alter the number of nigral dopaminergic neurons nor striatal dopamine levels. We then investigated the effect of overexpression of human alpha-synuclein in the substantia nigra of aged (18 to 21-month-old) L444P *Gba1* mice. Following intraparenchymal injections of human alpha-synuclein carrying viral vectors, pathological accumulation of phosphorylated alpha-synuclein occurred within the transduced neurons. Stereological counts of nigral dopaminergic neurons revealed a significantly greater cell loss in *Gba1*-mutant than wild-type mice. These results indicate that *Gba1* deficiency enhances neuronal vulnerability to neurodegenerative processes triggered by increased alpha-synuclein expression.

## Teaser

Mutations in glucocerebrosidase 1 (*GBA1*) are the commonest risk factor for Parkinson’s disease. Using *Gba1* mouse models, Migdalska-Richards *et al.* reveal an interaction between glucocerebrosidase activity and alpha-synuclein accumulation. *Gba1* deficiency increases vulnerability of nigral dopaminergic neurons to degeneration triggered by increased alpha-synuclein expression.

## Introduction

Parkinson’s disease is the second most common neurodegenerative disorder after Alzheimer’s disease and has been estimated to affect 4% of individuals by 80 years of age. Recently, both heterozygous and homozygous mutations in the glucocerebrosidase 1 (*GBA1*) gene have been linked to Parkinson’s disease, with 10–30% of individuals with *GBA1* mutations developing Parkinson’s disease by the age of 80 (Anheim *et al.*, 2012; Rana *et al.*, 2013; Alcalay *et al.*, 2014; Migdalska-Richards and Schapira, 2016). It is estimated that approximately 10–25% of Parkinson’s disease patients have a *GBA1* mutation (Parkinson-*GBA1*), with the most common mutations being L444P and N370S (Sidransky *et al.*, 2009; Bultron *et al.*, 2010; McNeill *et al.*, 2012*a*, *b*; Schapira, 2015). *GBA1* encodes glucocerebrosidase (GCase), a lysosomal enzyme that catabolizes glycolipid glucocerebroside to ceramide and glucose (Beutler, 1992). GCase activity is significantly decreased in different brain regions in Parkinson-*GBA1* patients. Moreover, significant reduction in GCase activity and protein levels is also observed in the brains of Parkinson’s disease patients without *GBA1* mutations (Gegg *et al.*, 2012; Murphy and Halliday, 2014), indicating the potential importance of GCase in the development of idiopathic Parkinson’s disease.

The clinical manifestation of Parkinson-*GBA1* is very similar to that for idiopathic Parkinson’s disease, but with a slightly younger age of onset, a higher incidence of neuropsychiatric features (including depression, anxiety, sleep disturbance and hallucinations) and a tendency to earlier and more prevalent cognitive impairment (Tan *et al.*, 2007; Neumann *et al.*, 2009; Sidransky *et al.*, 2009; Brockmann *et al.*, 2011; McNeill *et al.*, 2012*b*; Winder-Rhodes *et al.*, 2013). The pathology of Parkinson-*GBA1* is identical to that for idiopathic Parkinson’s disease, with the loss of dopaminergic neurons in the substantia nigra and the presence of Lewy bodies and neurites containing alpha-synuclein. Although the exact mechanism by which *GBA1* mutations increase Parkinson's risk is still unknown, it is likely that, as in idiopathic Parkinson’s disease, accumulation of alpha-synuclein, autophagic and lysosomal dysfunction, mitochondrial impairment, and oxidative and endoplasmic reticulum stress may all contribute to the development and progression of Parkinson-*GBA1* (Schapira and Tolosa, 2010; Sato *et al.*, 2013; Migdalska-Richards and Schapira, 2016). The central role of alpha-synuclein accumulation in the pathology of Parkinson-*GBA1* was further highlighted by the reciprocal relationship between GCase activity and alpha-synuclein; demonstrated in SH-SY5Y cell cultures, neuronal cultures, conduritol-β-epoxide (CBE)-treated mice and transgenic *Gba1* mouse models (Manning-Boĝ *et al.*, 2009; Cullen *et al.*, 2011; Mazzulli *et al.*, 2011; Sidransky and Lopez, 2012; Cleeter *et al.*, 2013; Osellame *et al.*, 2013; Sardi *et al.*, 2013; Schöndorf *et al.*, 2014; Xu *et al.*, 2014; Yang *et al.*, 2017). Conversely, it has also been demonstrated in cell and mouse models that increased alpha-synuclein causes a decrease in GCase activity (Gegg *et al.*, 2012; Migdalska-Richards *et al.*, 2016).

To date, several homozygous *Gba1* mouse models have been generated. These were originally used to study Gaucher's disease, a recessive lysosomal storage disorder related to GCase deficiency (Farfel-Becker *et al.*, 2009). However, recently some of these models have also been used for Parkinson’s studies with the intention of investigating the link between Gaucher's and Parkinson’s diseases. In a model where GCase deficiency was induced by single injections of CBE increased alpha-synuclein levels were observed after 48 h both in the cell bodies of the substantia nigra, and in the cytoplasm and cell nuclei of A9 neurons (Manning-Boĝ *et al.*, 2009). Another model, the homozygous *Gba1* mouse carrying either a D409H or V394L knock-in mutation together with a prosaposin hypomorphic transgene showed ∼80% reduction in GCase activity and a progressive accumulation of alpha-synuclein between 4 and 24 weeks of age in the brainstem, cortex, hippocampus, basal ganglia and some regions of the cerebellum. In addition, these knock-in mice also displayed accumulation of glucosylceramide and a progressive neurological decline. Similarly, the homozygous *Gba1* mice carrying a D409H knock-in mutation, but without the prosaposin hypomorphic transgene, showed reduced GCase activity and alpha-synuclein accumulation in the brainstem and cerebellum, but no neurological dysfunction (perhaps, because not all the same brain regions were affected) at 42 weeks of age (Xu *et al.*, 2011). The D409V knock-in mutation model showed progressive accumulation of proteinase K-resistant alpha-synuclein aggregates in the hippocampus between 2 and 12 months of age. These mice also showed elevated levels of glucosylsphingosine and impaired memory (Sardi *et al.*, 2011). However, the most severe phenotype amongst all homozygous *Gba1* mouse models was observed in mice carrying a knock-out *Gba1* mutation. These mice showed the most prominent reduction of GCase activity (10% residual), with increased oligomeric alpha-synuclein levels, accumulation of both glucosylceramide and glucosylsphingosine, and a rapid neurological decline leading to death by postnatal Day 14 (Enquist *et al.*, 2007; Osellame *et al.*, 2013).

Heterozygous *Gba1* mouse models have been less frequently studied. Analysis of heterozygous *Gba1* mice carrying a D409V knock-in mutation and heterozygous knock-out *Gba1* mice in both cases showed a similar reduction in GCase activity (∼50%), absence of glucosylceramide and glucosylsphingosine accumulation, and no memory impairment at 6 months of age. Interestingly, the D409V knock-in mice, but not the knock-out mice, showed the presence of protein K-resistant alpha-synuclein aggregates. However, the number of aggregates was only 50% of the level present in the same knock-in homozygous mice (Sardi *et al.*, 2011). Heterozygous *Gba1* mice carrying a L444P knock-in mutation in the presence of either wild-type human alpha-synuclein or human alpha-synuclein expressing the A53T mutation in the absence of endogenous mouse alpha-synuclein, displayed ∼40% reduction in GCase activity. Primary neuronal cultures obtained from these mice showed reduced degradation and increased accumulation of alpha-synuclein. Moreover, more rapid decline in motor and gastrointestinal function was observed in mice carrying both the murine L444P *Gba1* mutation and the human A53T alpha-synuclein mutation than in mice expressing only human A53T alpha-synuclein mutation (Fishbein *et al.*, 2014). Previous work from our group investigating heterozygous *Gba1* mice carrying a L444P knock-in mutation at 10–12 weeks of age showed that the reduction in GCase activity present in different brain regions could be alleviated (by ∼20%) by treatment with a molecular chaperone, ambroxol. The same study also demonstrated in different brain areas of human alpha-synuclein overexpressing mice, the reciprocal relationship between alpha-synuclein levels and GCase activity by showing that increase in GCase activity corresponded with about a 20% decrease in alpha-synuclein protein levels following ambroxol treatment (Migdalska-Richards *et al.*, 2016). Collectively, the data obtained from these studies clearly demonstrate the relationship between GCase deficiency and alpha-synuclein accumulation, and support the notion that *Gba1* mutations can cause Parkinson's-like alpha-synuclein pathology in mice.

Ageing is an unequivocal risk factor for Parkinson’s disease. In the present study, we used two aged (24-month-old) heterozygous *Gba1* mouse models (one carrying a *Gba1* knock-out mutation and one with a L444P *Gba1* knock-in mutation) to analyse the impact of *Gba1* mutations on behaviour, GCase activity, alpha-synuclein accumulation and dopaminergic neurodegeneration. For both models, we observed increased alpha-synuclein accumulation in the absence of other gross histopathological changes, and the absence of any major behavioural change related to Parkinson’s disease. We then analysed the effect of overexpression of human alpha-synuclein in the substantia nigra of aged (18 to 21-month-old) heterozygous *Gba1* mice carrying the L444P knock-in mutation. This overexpression, induced by an intraparenchymal injection of adeno-associated virus (AAV) carrying the *SNCA* gene, resulted in a greater loss of nigral dopaminergic neurons in *Gba1* than wild-type mice. Together these results suggest that *GBA1* mutations alone are not sufficient to cause overt Parkinson-like pathology, but that additional factors, such as overexpression of alpha-synuclein, are required for this pathology to develop.

## Materials and methods

### Mice

Mice were treated in accordance with local ethical committee guidelines and the UK Animals (Scientific Procedures) Act 1986. All procedures were carried out in accordance with Home Office guidelines (UK) and in compliance with the ARRIVE guidelines. B6;129S4-Gbatm1Rlp/Mmnc (000117-UNC) mice expressing heterozygous knock-in L444P mutation in the murine *Gba1* gene (L444P/+ mice) were purchased from the Mutant Mouse Regional Resource Centre (MMRRC); in these mice, a part of the *Gba1* gene is duplicated, but only the mutated copy is expressed (Liu *et al.*, 1998). Of note, these mice are wild-type for the glucosylceramide synthase (*Ugcg*) gene, and have an intact adjacent downstream metaxin gene. Transgenic mice containing heterozygous L444P mutation were identified by PCR of ear plug genomic DNA using forward primer 5′TGTGAAGTTCCTGGATGCCTATG-3’ and reverse primer 5′TGGTGATGTCTACAATGATGGGAC-3’. K14-lnl/+ mice expressing heterozygous knock-out mutation in the murine *Gba1* gene (*KO/+* mice) were obtained from the laboratory of Professor Karlsson (Enquist *et al.*, 2007). Transgenic mice containing heterozygous knock-out mutation were identified by PCR of ear plug genomic DNA using forward primer 5′-GTACGTTCATGGCATTGCTGTTCACT-3’ and reverse primer 5'-AAGACAGAATAAAACGCACGGGTGTTGG-3'. Both *L444P*/+ and *KO*/+ mice were maintained on a mixed C57BL/G, 129Sv, FVB background. Only male animals were used for most of the study; both male and female mice were used for experiments in which animals were injected with human alpha-synuclein-AAV. Animals were usually culled at 24 months of age, except for mice injected with human alpha-synuclein-AAV that were culled between 18 to 21 months of age.

### Enzyme assays

GCase and β-hexosaminidase activities were measured as described before (Migdalska-Richards *et al.*, 2016). For both assays, five *L444P/+* mice, six *KO/+* mice, six wild-type littermates for *L444P/+* mice and seven wild-type littermates for *KO/+* mice were analysed for each region.

### Immunohistochemistry

Mice were killed by CO_2_ inhalation, brains were extracted and immediately placed in 10% neutral-buffered formalin. Brains were kept in formalin for at least 2 weeks and then paraffinized. Paraffinized brains were sliced sagittally into 12 µm sections using a sledge microtome. Sections were stretched by floating them on water at 45°C, and collected on SuperFrost® Plus microscope slides (Thermo Scientific). Prior to immunohistochemistry, the sections were incubated on slides at 65°C overnight, deparaffinized in xylene (Thermo Fisher Scientific) for 10 min and rehydrated by sequential incubations in 100%, 95%, 70% and 50% ethanol (Sigma-Aldrich) then in water and in phosphate-buffered saline (PBS, Life Technologies) for 10 min each. Antigen retrieval was performed for alpha-synuclein and ionized calcium binding adaptor molecule 1 (Iba1) staining, by incubating the sections in 10 mM sodium citrate (Sigma-Aldrich), pH 8.5, at 80°C for 30 min; for antigen retrieval of glial fibrillary acidic protein (GFAP), sections were incubated in proteinase K solution (Sigma-Aldrich) in Tris-EDTA (TE) buffer (50 mM Tris base, 1 mM EDTA, 0.5% Triton™ X-100, pH 8.0), 20 µg/ml, at room temperature for 5 min. Proteinase K activity was blocked by incubation adding 5 mM phenylmethylsulfonyl fluoride (PMSF; Sigma-Aldrich). Sections were then washed with PBS + 0.3% Triton™ X-100 (PBST) (Sigma-Aldrich), incubated in 20% methanol + 3% H_2_O_2_ (Sigma-Aldrich) in PBST at room temperature for 1 h (to inhibit the activity of endogenous peroxidases) and non-specific background blocked by incubation with 5% normal horse serum (NHS) (Vector Laboratories) in PBST at room temperature for 1 h. Sections were then incubated with primary antibodies diluted in PBST at 4°C overnight. The following antibodies were used: rabbit anti-αSYN (Cell Signaling D37A6, 1:500), rabbit anti-TH (Abcam ab112, 1:500), rabbit anti-Iba1 (Wako Chemicals, 1:400), and rabbit anti-GFAP (Dako Z0334, 1:500). Following 30 min wash in PBST, sections were incubated with secondary horse anti-rabbit biotinylated antibody (Vector Laboratories) diluted at 1:2000 in PBST + 5% normal horse serum at room temperature for 3 h followed by avidin/biotin Vectastain Elite ABC HRP Kit (Vector Laboratories) according to the manufacturer’s instructions. Staining was developed using DAB Peroxidase Substrate Kit (Vector Laboratories) and sections were counterstained with 0.1% cresyl violet (Sigma-Aldrich) in 0.3% acetic acid for 5 min at room temperature, dehydrated in ascending ethanol series, defatted in xylene, coverslip mounted with DPX Mountant (Sigma-Aldrich). Staining was analysed using Olympus BX53 microscope. To increase the specificity of immunohistochemical detection of insoluble alpha-synuclein, the sections were pretreated with proteinase K. Briefly, deparaffinized sections were washed with PBS, incubated for 10 min with proteinase K at 20 μg/ml in PBST at 37°C and then incubated twice for 5 min with 5 mM PMSF in PBST to inhibit the activity of proteinase K. Next, the sections were washed with PBST and processed as described above for immunohistochemical detection of alpha-synuclein antibody, D37A6.

For each immunohistochemical staining, three *L444P/+* mice, three *KO/+* mice, three wild-type littermates for *L444P/+* mice and three wild-type littermates for *KO/+* mice were analysed for each region.

### Western blotting

Brain samples were homogenized in 5 mM EDTA, 750 mM sodium chloride, 50 mM Tris (pH 7.4), 10% Triton™ X-100, 1× Halt Protease Inhibitor Cocktail (Thermo Scientific) and 1× Halt Phosphatase Inhibitor Cocktail (Thermo Scientific). To detect total alpha-synuclein levels, brain samples were homogenized in 10 mM Tris (pH 7.4), 0.1% sodium dodecyl sulphate, 1× Halt Protease Inhibitor Cocktail (Thermo Scientific) and 1× Halt Phosphatase Inhibitor Cocktail (Thermo Scientific) and incubated at 37°C for at least 1 h*.* Respective homogenates were centrifuged to remove insoluble materials and protein concentrations were determined using a Pierce BCA Protein Assay (Thermo Scientific). Supernatants were supplemented with distilled water to contain 30 μg protein in a total volume of 15 µl (samples consisted of least 75% of supernatant). Next, 1 µl of NuPAGE® sample reducing agent (Thermo Scientific) and 4 µl of NuPAGE® LDS sample buffer (Thermo Scientific) were added to each sample. Respective samples were incubated at 70°C for 10 min before being separated on 4–12% or 12% NuPAGE® Tris-Bis gels (Invitrogen). The gels were subsequently transferred to Hybond-P membranes (GE Healthcare). The transferred membranes were cut at just above 20 kDa band, and the bottom parts of the membranes were fixed with 4% paraformaldehyde (Sigma-Aldrich) containing 0.001% glutaraldehyde (Sigma-Aldrich) for 30 min, and then washed with 1% PBS + 1% Tween 20 (Sigma-Aldrich) 3× for 5 min. Next, both fixed and non-fixed parts of the membranes were blocked in 5% skim milk in PBS-Tween 20 for 1 h. Membranes were incubated with primary antibodies (see Supplementary material for the list of primary antibodies), and then with the respective secondary antibodies (Dako). Bands were detected by Pierce ECL Western Blotting Substrate (Thermo Scientific) or Luminata Forte Western HPR Substrate (Millipore). Band intensity was measured using the ChemiDoc MP System, Biorad. Protein expression was expressed as a ratio against β-actin.

For each western blotting, five *L444P/+* mice, five *KO/+* mice, five wild-type littermates for *L444P/+* mice and five wild-type littermates for *KO/+* mice were analysed for each region.

### Adeno-associated virus induced overexpression of human alpha-synuclein

#### Vector

Recombinant AAVs (serotype 2 genome packaged in serotype 6 capsid) were used for transgene expression of human wild-type alpha-synuclein. Gene expression was under the control of the human synapsin 1 promoter and was enhanced using a woodchuck hepatitis virus post-transcriptional element (WPRE) and a polyadenylation signal sequence (Loeb *et al.*, 1999). AAV production and titration were performed by Vector Biolabs (Malvern) (Ulusoy *et al.*, 2013, 2015; Helwig *et al.*, 2016).

#### Stereotactic injection

Human alpha-synuclein-AAV preparations were diluted and injected at a titre of 10^13^ genome copies/ml. Surgical procedures were performed under isoflurane anaesthesia. Mice (16 to 19 months of age) were placed in a stereotactic head frame (Stoelting). After making a midline incision of the scalp, a hole was drilled on the right side of the skull. Each animal received a single intraparenchymal injection into the right ventral mesencephalon immediately dorsal to the substantia nigra pars compacta at the following coordinates: −2.4 mm antero-posterior and −1.1 mm medial-lateral relative to bregma, and −4.2 mm dorsal-ventral from the surface of the dura (Paxinos and Franklin, 2001). An amount of 1.5 µl of AAV preparation was injected at a speed of 0.5 µl/min with a Hamilton syringe fitted with a glass capillary needle. The needle was left in place for five additional minutes before its slow retraction.

#### Tissue preparation following adeno-associated virus injection

Mice were sacrificed by cervical dislocation 2 months after the stereotactic injections. Brains were removed and striata quickly dissected on ice. Tissue specimens were placed in ice-cold 0.4 M perchloric acid, homogenized by sonication and centrifuged at 15 000*g* for 10 min. The supernatant was filtered through a 0.22 µm membrane and used for determination of striatal dopamine (DA) and 3,4-dihydroxyphenylacetic acid (DOPAC) concentrations.

The remaining brain tissue was post-fixed in 4% (w/v) paraformaldehyde for 48 h and then cryopreserved in 25% (w/v) sucrose solution. Coronal sections (40 µm) were cut using a freezing microtome and stored at −20°C in phosphate buffer (pH 7.4) containing 30% glycerol and 30% ethylene glycol.

High performance liquid chromatography measurement. Dopamine and DOPAC concentrations were measured by reverse phase high performance liquid chromatography coupled with electrochemical detection (Coulochem III, Thermo Scientific), as described by Kilpatrick and colleagues (1986). Dopamine and DOPAC were separated using a C18 reverse phase column (Thermo Scientific) at a flow rate of 0.6 ml/min. The mobile phase consisted of 10% acetonitrile, 75 mM NaH_2_PO_4_·H_2_O, 0.17 mM octanesulphonic acid, 2.5 mM triethylamine and 25 mM EDTA, adjusted to pH 3.0 with orthophosphoric acid. Catecholamine concentrations are expressed per milligram protein. Dopamine and DOPAC concentrations were measured in six *L444P/+* and three wild-type mice.

#### Immunohistochemistry

Immunohistochemistry was performed on free-floating sections. Samples were rinsed in Tris buffer (TBS, pH 7.6), quenched for 1 h in 3% H_2_O_2_ / 10% methanol in TBS, and blocked for 1 h in 5% normal serum in TBS containing 0.25% Triton™ X-100 (TBS-T). Sections were first incubated with a monoclonal rat primary antibody against human alpha-synuclein (Enzo Life Science ALX-805-258-L001, 1:2000) or a polyclonal rabbit primary anti-TH antibody (Millipore AB152, 1:2000) at room temperature overnight or for 36 h, respectively. They were then incubated with a species-specific secondary antibody (Vector Laboratories) for 2 h at room temperature. Following treatment with streptavidin-horseradish peroxidase complex (ABC Elite kit, Vector Laboratories), alpha-synuclein and TH immunoreactivities were visualized using 3,3′-diaminobenzidine kit (Vector Laboratories). Sections were finally mounted on coated slides, dried, stained with cresyl violet (only TH staining) (FD Neurotechnologies) and cover-slipped with Depex (Sigma-Aldrich). To immunostain for phosphorylated alpha-synuclein, a few modifications were made. Sections were quenched for 30 min in Bloxall blocking solution (Vector Laboratories SP-6000), and blocked for 1 h in 2.5% normal horse serum in TBS-T. Incubations with monoclonal rabbit primary antibody against human alpha-synuclein phosphorylated at S129 (Abcam 51253, 1:10 000) was followed by treatment with ImmPRESS Reagent anti-rabbit Ig (Vector Laboratories MP-7401) for 1 h at room temperature. For fluorescence microscopy, tissue sections were incubated with a monoclonal rat primary antibody against human alpha-synuclein (Enzo Life Science ALX-805-258-L001, 1:750) together with a polyclonal rabbit primary anti-TH antibody (Millipore AB152, 1:2000) at room temperature overnight. Immunoreactivity was visualized with secondary antibodies conjugated with the fluorophores Alexa Fluor® 594 (Thermo Fisher Scientific A11007, 1:300) or DyLight® 488 (Vector Laboratories DI-1088, 1:300). Fluorescent labelled sections were mounted on coated slides and cover-slipped with Vectashield Hard Set Mounting Medium (Vector Laboratories H-1400). Images were acquired using a LSM 700 Zeiss upright confocal microscope equipped with 488 and 555 nm excitation lasers. Stack images were collected at 8 μm interval with a 10× objective and at 0.6 μm with a 63× objective. Single images were then generated using maximum intensity projection postprocessing. For each immunohistochemical staining, three *L444P/+* and three wild-type mice were analysed.

#### Cell counting

To estimate the number of nigral dopaminergic neurons, stereological counts were performed by investigators blind to the experimental groups. The total number of TH-immunoreactive or Nissl-stained neurons was estimated in the substantia nigra pars compacta using the optical fractionator (Stereo Investigator software version 9, MBF Biosciences). Every fifth section throughout the whole substantia nigra was sampled. After delineation of the substantia nigra pars compacta with a 4× objective, counts were performed at ×60 magnification using the following parameters: 1 µm guard zone, 60 × 60 mm counting frame, 125 × 125 mm sampling grid size. Coefficient of error was calculated according to Gundersen and Jensen (1987) and values <0.10 were accepted. To estimate the percentage of TH nigral neurons expressing human alpha-synuclein, single stack images of double-immunostained midbrain section were analysed. In these sections, the total number of TH-positive cells was counted together with the number of TH-immunoreactive neurons that were also labelled for human alpha-synuclein. Cell counts were performed in four *L444P/+* and four *KO/+* mice.

### Behavioural tests

Behavioural tests to assess olfaction (buried pellet test), coordination (pole test) and cognition (novel object recognition test) were conducted on mice that were 22 months old and were group housed from weaning. See Supplementary material for a detailed description of the tests.

### Statistical analysis

Data are expressed as mean ± SEM (standard error of the mean) and statistical significance between groups were analysed with the unpaired *t*-test or one-way analysis of variance (ANOVA), followed by the Tukey honestly significant difference (HSD) test.

## Results

### Reduced glucocerebrosidase activity in *Gba1* mutant mice

**Figure 1:**
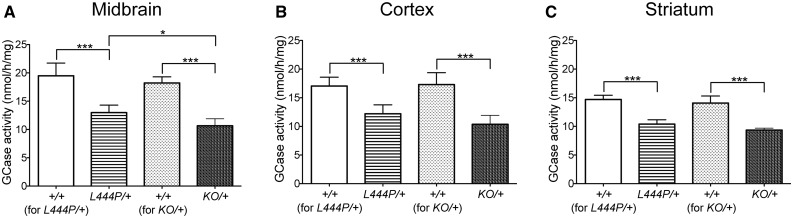
**Glucocerebrosidase (GCase) enzyme activity in *L444P/+* and *KO/+* mouse brains.** (**A**–**C**) GCase activity was significantly decreased in the midbrain, cortex and striatum of *L444P/+* mice (*n* = 5) and KO/+ mice (*n* = 6) compared to their corresponding wild-type (*+/+*) control littermates (*n* = 6 and *n* = 7, respectively). Data were analysed with the one-way ANOVA test, followed by *post hoc* analysis using the Tukey HSD test. *P < 0.05, **P ≤ 0.01, ***P ≤ 0.001.

To determine GCase activity in aged *L444P/+* and *KO/+* mice, and to investigate whether heterozygous knock-in and knock-out mutations in the *Gba1* gene have the same effect on GCase activity, GCase activity was measured in the midbrain, cortex and striatum of *L444P/+* and *KO/+* mice and their corresponding wild-type control littermates. The one-way ANOVA analysis showed a statistically significant difference in GCase activity between *L444P/+*, *KO/+* and wild-type control mice in the midbrain [*F*(3,21) = 53.62, *P* < 0.0001], cortex [*F*(3,19) = 24.82, *P* < 0.0001] and striatum [*F*(3,19) = 51.23, *P* < 0.0001]. The *post hoc* analysis using the Tukey HSD test showed that GCase activity was significantly decreased in midbrain (31%), cortex (28%) and striatum (28%) of *L444P/+* mice compared to their wild-type control littermates, and in the midbrain (41%), cortex (42%) and striatum (34%) of *KO/+* mice compared to wild-type control littermates (Table 1 and Fig. 1A–C). The *post hoc* analysis using the Tukey HSD test also revealed that GCase activity was significantly decreased (18%) in the midbrain of *KO/+* mice compared to *L444P/+* mice (Table 1 and Fig. 1A). GCase activity was also decreased in the cortex (15%) and striatum (10%) of *KO/+* mice compared to *L444P/+* mice, but these changes did not reach statistical significance (Table 1, Fig. 1B and C). To determine whether GCase deficiency impacts lysosomal content, β-hexosaminidase activity was measured in the midbrain, cortex and striatum of *L444P/+* and *KO/+* mice and their corresponding wild-type control littermates, but no statistically significant changes were observed.

**Table 1:** Decrease in GCase activity in *Gba1* mutant mice

Brain region	Mouse model		
	*L444P/+*	*KO/+*	*KO/+* versus *L444P/+*
Midbrain	*31%	*41%	*18%
Cortex	*28%	*42%	15%
Striatum	*28%	*34%	10%

*Statistically significant decrease.

### Prominent increase in alpha-synuclein accumulation in multiple regions of *L444P/+* and *KO/+* mice

**Figure 2:**
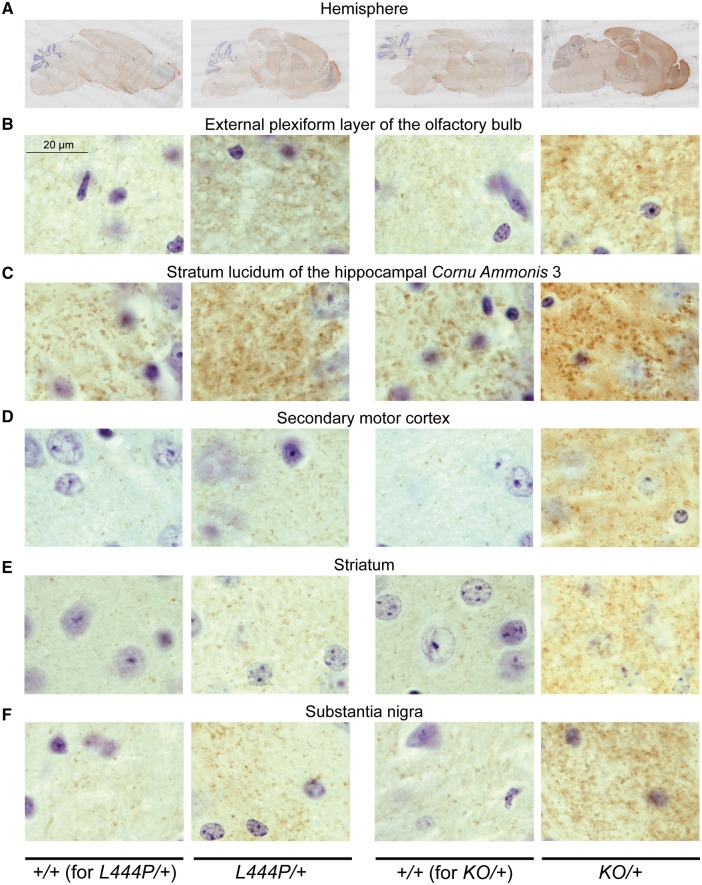
**Alpha-synuclein immunohistochemistry in *L444P/+* and *KO/+* mouse brains.** (**A**) The entire hemisphere in *L444P/+* and *KO/+* mice and their corresponding wild-type (*+/+*) control littermates. (**B**–**F**) Increased alpha-synuclein staining in the external plexiform layer of olfactory bulb (**B**), stratum lucidum of the hippocampal cornu ammonis 3 (CA3) region (**C**), secondary motor cortex (**D**), striatum (**E**) and substantia nigra (**F**) of *L444P/+* and *KO/+* mice compared to their corresponding wild-type control littermates. Scale bars = 20 μm. Representative images are shown. Three mice of each genotype were analysed.

**Figure 3:**
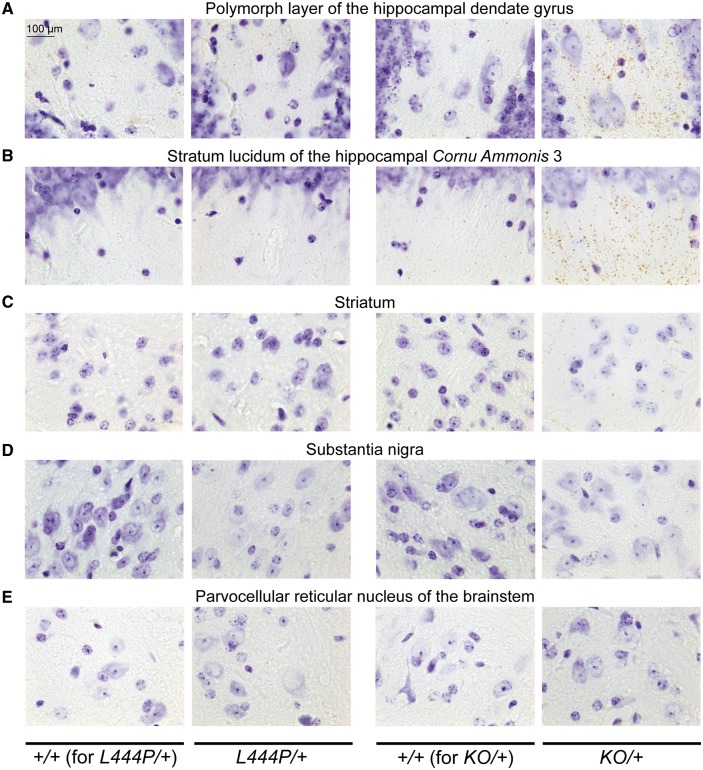
**Alpha-synuclein immunohistochemistry after proteinase K treatment in *L444P/+* and *KO/+* mouse brains.** (**A**–**C**) Accumulation of small alpha-synuclein proteinase K-resistant aggregates in the polymorph layer of the hippocampal dendate gyrus (**A**), stratum lucidum of the hippocampal cornu ammonis 3 (CA3) region (**B**) and striatum (**C**) of *KO/+* mice, but not *L444P/+* mice compared to their corresponding wild-type (*+/+*) control littermates. (**D**–**E**) No proteinase K-resistant alpha-synuclein aggregates were found in the substantia nigra (**D**) and parvocellular reticular nucleus of the brainstem (**E**) of *KO/+* and *L444P/+* mice compared to their corresponding wild-type control littermates. Scale bars = 100 μm. Representative images are shown. Three mice of each genotype were analysed.

To investigate the relationship between GCase activity and alpha-synuclein levels, alpha-synuclein immunohistochemistry was performed in *L444P/+* and *KO/+* mice and their corresponding wild-type control littermates. An overall increase in alpha-synuclein staining was observed in brains of both *L444P/+* and *KO/+* animals (Fig. 2A). Detailed analysis revealed a punctate distribution of alpha-synuclein in the analysed brain regions in wild-type control animals that was consistent with synaptic and/or vesicular enrichment of this protein in physiological conditions. Although similar distributions were found in both *L444P/+* and *KO/+* mice, more noticeable and, in some cases, larger alpha-synuclein-positive puncta were observed in *KO/+* mice (Fig. 2A–F). Prominent increase in alpha-synuclein staining was observed in the external plexiform layer of the olfactory bulb, the stratum lucidum of the cornu ammonis 3 (CA3) region of the hippocampus, the secondary motor cortex, the striatum and the substantia nigra of both *L444P/+* and *KO/+* mice compared to their corresponding wild-type control littermates (Fig. 2B–F). Substantial increase in alpha-synuclein staining was also observed in the anterior area of the olfactory bulb and the polymorph layer of the hippocampal dentate gyrus, while a less prominent increase was detected in the stratum oriens of the hippocampal CA1 region, the retrosplenial cortex and the brainstem of both *L444P/+* and *KO/+* mice. Next, selected regions of the brain were analysed for the presence of proteinase K-resistant alpha-synuclein aggregates. Proteinase K pretreatment prior to alpha-synuclein immunohistochemistry revealed accumulation of small aggregates in the hippocampus (especially significant in the stratum lucidum of the CA3 region and in the polymorph layer of the dentate gyrus), and to lesser extend in the striatum of *KO/+* mice (Fig. 3A–C). No proteinase K-resistant aggregates were found in the substantia nigra and parvocellular reticular nucleus of the brainstem of *KO/+* mice (Fig. 3D and E). Also, no proteinase K-resistant alpha-synuclein aggregates were detected in any of the analysed regions of *L444P/+* mice (Fig. 3A–E).

**Figure 4:**
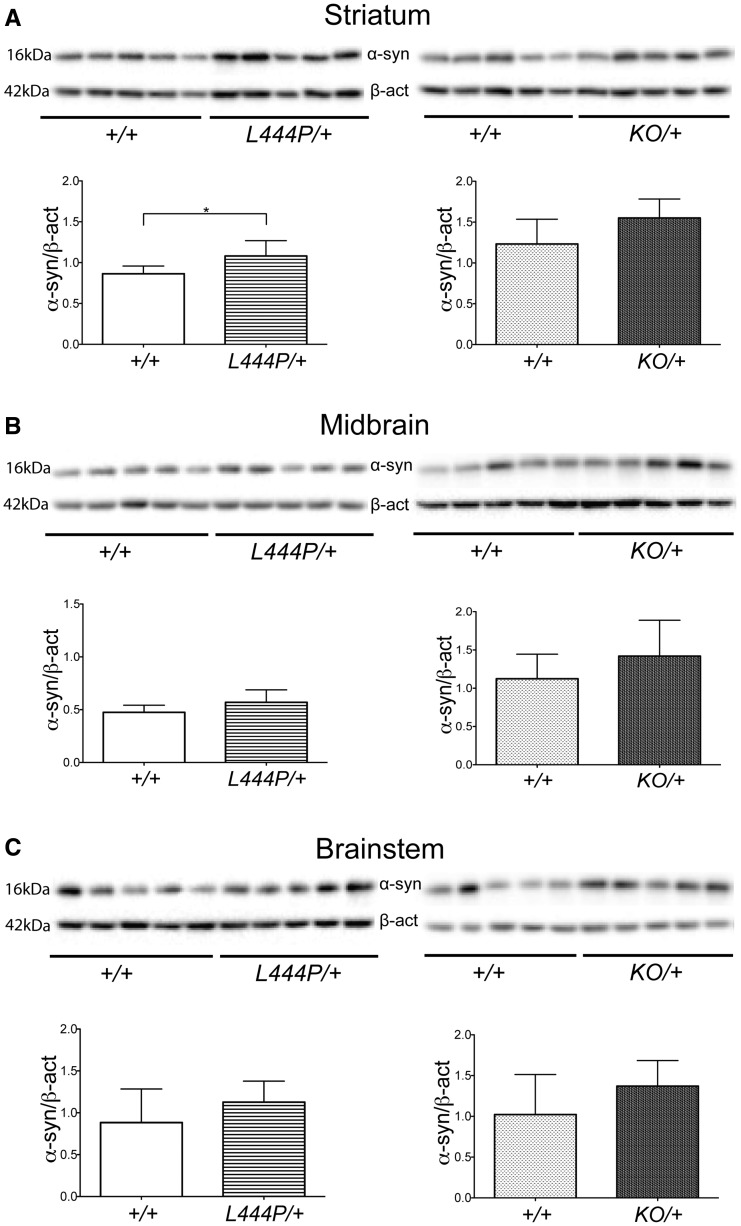
**Alpha-synuclein protein levels in *L444P/+* and *KO/+* mouse brains.** (**A**) Western blotting for alpha-synuclein in the striatum. Alpha-synuclein protein levels were significantly increased in the striatum of *L444P/+* mice (*n* = 5) compared to wild-type (*+/+*) control mice (*n* = 5). Alpha-synuclein protein levels were also increased in the striatum of *KO/+* mice (*n* = 5) compared to wild-type control mice (*n* = 5), but this was not statistically significant. (**C**) Western blotting for alpha-synuclein in the midbrain. Alpha-synuclein protein levels were increased in the midbrain of *L444P/+* mice (*n* = 5) compared to wild-type control mice (*n* = 5), but this increase did not reach statistical significance. Alpha-synuclein protein levels were increased in the midbrain of *KO/+* mice (*n* = 5) compared to wild-type control mice (*n* = 5), but again without statistical significance. (**C**) Western blotting for alpha-synuclein in the brainstem. Alpha-synuclein protein levels were increased in the brainstem of *L444P/+* mice (*n* = 5) compared to wild-type control mice (*n* = 5), but this was not statistically significant. Alpha-synuclein protein levels were also increased in the brainstem of *KO/+* mice (*n* = 5) compared to wild-type control mice (*n* = 5), but this increase also did not reach statistical significance. Data were analysed with the unpaired *t*-test. *P < 0.05 versus control, **P ≤ 0.01 versus control, ***P ≤ 0.001 versus control.

To determine the level of alpha-synuclein increase in *L444P/+* and *KO/+* mice, alpha-synuclein protein levels were measured by western blotting analysis in the striatum, midbrain and brainstem. Alpha-synuclein levels were significantly increased in the striatum of *L444P/+* mice compared to wild-type control littermates (25%; unpaired *t*-test *P* = 0.0491) (Fig. 4A). Alpha-synuclein protein levels were also increased in the midbrain and brainstem of *L444P/+* mice compared to wild-type control littermates, but these changes did not reach statistical significance (Fig. 4B and C). Alpha-synuclein protein levels were elevated in the striatum, midbrain and brainstem of *KO/+* mice compared to wild-type control littermates, but again none of these changes reached statistical significance (Fig. 4A–C). Prominent increase in alpha-synuclein staining in the striatum, substantia nigra and brainstem was not consistently replicated by western blotting, likely reflecting differences in sensitivity and tissue resolution between the two techniques. In particular, robust changes in punctate alpha-synuclein staining by immunohistochemistry appeared to result in relatively modest increases in total alpha-synuclein levels measured by western blotting. We measured the levels of phosphorylation of alpha-synuclein at serine 129 (S129) in the striatum, midbrain and brainstem of *L444P/+, KO/+* mice and their corresponding wild-type control littermates by western blotting. No significant changes in S129 phosphorylation of alpha-synuclein were observed in any brain region or mouse model.

### Absence of other pathological hallmarks related to Parkinson’s disease in *L444P/+* and *KO/+* mice

Immunohistochemical analysis of tyrosine hydroxylase (TH), ionized calcium binding adaptor molecule 1 (Iba1) and glial fibrillary acidic protein (GFAP) proteins revealed no evident changes in *L444P/+* and *KO/+* mice compared to their wild-type control littermates (Supplementary Figs 1–3), except for an increased number of Iba1-positive microglial cells in the granule cell layer of the olfactory bulb in *L444P/+* mice (Supplementary Fig. 4). In addition to TH, Iba1 and GFAP immunohistochemistry, a range of different proteins involved in processes that may be impaired in Parkinson-*GBA1* (such as autophagy, lysosomal functioning and endoplasmic reticulum stress) were analysed by western blotting. Cathepsin D, lysosome-associated membrane protein 1 (LAMP1), p62 (also known as sequestosome 1), microtubule-associated protein 1A/1B-light chain (LC3), binding immunoglobulin protein (BiP) and GFAP levels were measured in the striatum, midbrain and brainstem. No significant changes in levels of any of these proteins were observed in any analysed region of *L444P/+* and *KO/+* mice compared to their wild-type control littermates (Supplementary Tables 1 and 2).

### No olfactory, motor or cognitive impairment in *L444P/+* and *KO/+* mice

No statistically significant differences in the olfaction analysed by buried pellet test, coordination studied by pole test or cognition evaluated by novel object recognition test were observed in either *L444P/+* or *KO/+* mice compared to their corresponding wild-type control littermates (Supplementary Table 3).

### Enhanced dopaminergic cell loss after overexpression of human alpha-synuclein in *L444P/+* mice

**Figure 5:**
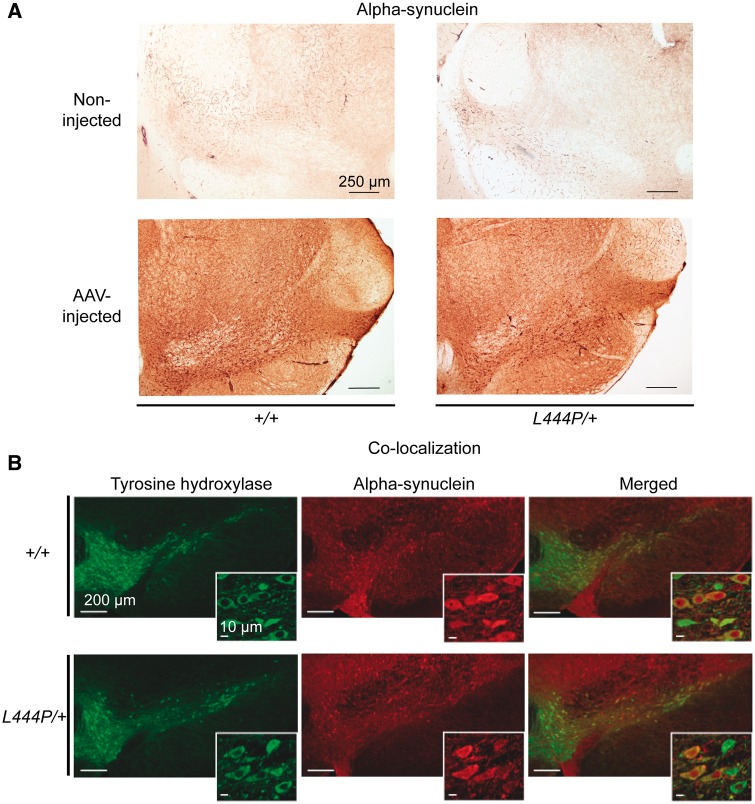
**Alpha-synuclein and tyrosine hydroxylase immunohistochemistry in *L444P/+* and wild-type (*+/+*) mice injected with human alpha-synuclein-carrying AAVs.** (**A**) Increased alpha-synuclein immunostaining in the injected versus non-injected ventral mesencephalon (including the substantia nigra) of *L444P/+* and wild-type control mice. Scale bars = 250 μm. Representative images are shown. Three mice of each genotype were analysed. (**B**) Co-labelling of the right (AAV-injected) ventral mesencephalon of *L444P/+* or wild-type mice with antibodies against tyrosine hydroxylase (TH) (green) and human alpha-synuclein (red) showed that the vast majority of dopaminergic neurons expressed the exogenous protein. Higher magnification images are displayed as *insets*. Scale bars = 200 μm (large images) and 10 μm (*insets*). Representative images are shown. Three mice of each genotype were analysed.

To test the hypothesis that a *Gba1* deficiency may enhance neuronal vulnerability to alpha-synuclein-induced pathology, *L444P/+* mice and their wild-type control littermates received a single intraparenchymal injection of human alpha-synuclein-AAV into the right ventral mesencephalon immediately dorsal to the substantia nigra pars compacta. The animals were sacrificed 2 months after AAV treatment. Immunohistochemical evaluation of midbrain sections labelled with a specific anti-human alpha-synuclein antibody revealed a light background staining on the unlesioned (left) side of the brain. In contrast, labelling was robust in the AAV-injected (right) midbrain of both *L444P/+* and wild-type control mice, confirming transgene expression (Fig. 5A). Following these intraparenchymal AAV injections, transgene expression was seen in the substantia nigra pars compacta as well as within neuronal populations near the administration site, including neurons in the substantia nigra pars reticulata and part lateralis, the ventral tegmental area, the mesencephalic reticular formation and posterior thalamic nuclei (Kirik *et al.*, 2002; Ulusoy *et al.*, 2017). Co-labelling with antibodies against TH and human alpha-synuclein confirmed that, within the substantia nigra pars compacta, the vast majority of dopaminergic neurons expressed the exogenous protein (Fig. 5B). Neuronal counts determined that 91.2(± 4.7) and 90.2 (± 2.2)% of TH-positive neurons were also immunoreactive for human alpha-synuclein in wild-type control and *L444P/+* mice, respectively.

**Figure 6:**
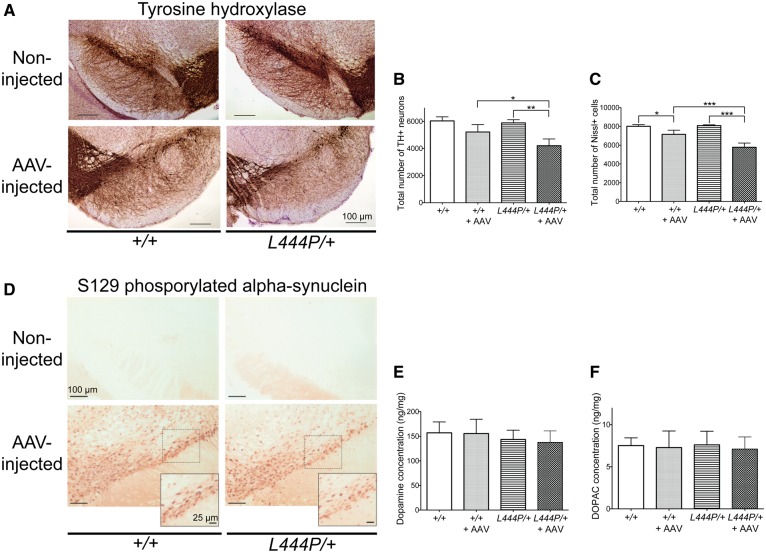
**Effects of AAV-induced alpha-synuclein transduction on the nigrostriatal system of *L444P/+* and wild-type (*+/+*) mice.** (**A**) Decreased TH immunostaining in the injected substantia nigra compared to the non-injected substantia nigra. The decrease in TH immunoreactivity was more prominent in the injected substantia nigra of *L444P/+* than wild-type control mice. Representative images are shown. Three mice of each genotype were analysed. (**B**) Stereological count revealed that the total number of TH-stained (TH+) neurons was significantly decreased in the right (AAV-injected) versus the left (non-injected) substantia nigra of *L444P/+* mice (*n* = 4). The total number of TH-stained neurons was also significantly decreased in the injected substantia nigra of *L444P/+* mice compared to the injected substantia nigra of wild-type control mice (*n* = 4). (**C**) Stereological count showed that the total number of Nissl-stained (Nissl+) cells was significantly decreased in the injected (*n* = 4) versus non-injected (*n* = 3) substantia nigra of *L444P/+* mice and wild-type control mice. The total number of Nissl-stained cells was also significantly decreased in the injected substantia nigra of *L444P/+* versus wild-type mice. (**D**) Accumulation of S129 phosphorylated alpha-synuclein in the right (AAV-injected) ventral mesencephalon of *L444P/+* and wild-type mice. Only background immunoreactivity was observed on the non-injected (*left*) side of the brain. The dashed square boxes encompass areas of the substantia nigra pars compacta that are shown at higher magnification. Scale bars = 100 μm (large images) and 25 μm (*insets*). Representative images are shown. Three mice of each genotype were analysed. (**E**) High performance liquid chromatography analysis showed no statistically significant changes in dopamine content in the striatum from the injected hemisphere of *L444P/+* or wild-type mice (*n* = 6 and *n* = 3, respectively) compared to the striatum from the corresponding non-injected hemisphere (*n* = 6 and *n* = 3, respectively). (**F**) High performance liquid chromatography analysis showed no statistically significant changes in DOPAC content in the striatum from the injected hemisphere of *L444P/+* or wild-type mice compared to the striatum from the non-injected hemisphere.

We then investigated the impact of human alpha-synuclein overexpression on the survival of nigral dopaminergic neurons by immunohistochemistry and stereological cell counting and compared it in control and transgenic mice. When midbrain sections were stained with an anti-TH antibody, the density of labelled nigral cells was evidently decreased on the right side of the brain of both *L444P/+* and wild-type control mice as a result of AAV transduction (Fig. 6A). Interestingly, a greater loss of TH-immunoreactive neurons was apparent in the right substantia nigra of *L444P/+* mice compared to wild-type controls (Fig. 6A). Stereological counting confirmed and extended these observations. The number of TH-immunoreactive neurons was unchanged in the unlesioned (left) substantia nigra pars compacta of *L444P/+* and wild-type control mice, indicating that *Gba1* deficiency did not itself induce any dopaminergic cell loss (Fig. 6B). On the other hand, overexpression of human alpha-synuclein in the AAV-injected (right) substantia nigra caused a slight decrease (−13%) in TH-positive cells in wild-type mice and a more pronounced reduction (−29%) in *L444P/+* animals (Fig. 6B). One-way ANOVA indicated a statistically significant difference between groups [*F*(3,11) = 14.67, *P* = 0.0004]. *Post hoc* analysis using the Tukey HSD test showed that the difference in counts of TH-immunoreactive neurons in the unlesioned (left) versus AAV-injected (right) substantia nigra reached statistical significance in *L444P/+* but not wild-type control mice. Importantly, the difference in neuronal number between the lesioned (right) substantia nigra of wild-type controls and *L444P/+* mice was also statistically significant, underscoring a greater vulnerability of *Gba1*-deficient mice to neurotoxicity triggered by human alpha-synuclein overexpression.

A decreased count of TH-positive neurons, as described above, could reflect cell death but could also be consequence of a downregulation of the phenotypic marker (i.e. TH) used for cell identification. To discriminate between these two possibilities, the total number of Nissl-stained neurons was also determined and compared in the left and right substantia nigra pars compacta of wild-type controls and *L444P/+* mice (Fig. 6C). Data analysis revealed a statistically significant difference between groups [ANOVA; *F*(3,11) = 36.79, *P* < 0.0001], and the Tukey HSD test indicated that the number of Nissl-stained cells was significantly decreased in the lesioned (right) versus unlesioned (left) substantia nigra of either wild-type control or *L444P/+* mice. The extent of this decrease was significantly greater in the latter (−29%) than the former (−11%) group of animals, confirming a synergistic effect of enhanced alpha-synuclein expression and *Gba1* deficiency on nigral neuronal death (Fig. 6C).

Hyperphosphorylated alpha-synuclein is a marker of pathological alpha-synuclein in the brain of Parkinson’s disease patients as well as Parkinson’s disease animal models (Fujiwara *et al.*, 2002; Luk *et al.*, 2012). Midbrain sections from wild-type control and *L444P/+* mice were immunostained with an antibody that specifically recognizes alpha-synuclein phosphorylated at S129. Only background staining was detected on the left (unlesioned) side of the brain. In contrast, robust immunoreactivity characterized neurons in the right (AAV-injected) mesencephalon of both wild-type and *L444P/+* animals (Fig. 6D). The density of labelled neurons appeared slightly decreased in the right substantia nigra pars compacta of *L444P/+* as compared to wild-type mice, most likely reflecting the more pronounced neurodegeneration triggered by alpha-synuclein overexpression in *Gba1*-deficient animals and a consequent greater loss of phosphorylated alpha-synuclein-containing cells (Fig. 6D).

In a final set of analyses, levels of dopamine and its metabolite DOPAC were measured in the striatum of wild-type control and *L444P/+* mice as markers of nigrostriatal dopaminergic function. *Gba1* deficiency alone or in combination with nigral overexpression of human alpha-synuclein did not affect striatal dopamine and DOPAC content that remained unchanged between wild-type control versus *L444P/+* mice. No difference was also present when data were compared in the uninjected (left) versus AAV-injected (right) side of the brain (Fig. 6E and F).

## Discussion

This study provides the first comprehensive behavioural and histochemical analysis of aged (24-month-old) heterozygous *Gba1* mice carrying (i) a knock-out mutation; or (ii) a L444P knock-in mutation. Using these models, we explored the link between *Gba1*-deficiency, alpha-synuclein overexpression and neurodegeneration in the substantia nigra. Altogether, our results provide novel insights into mechanisms by which *GBA1* mutations may enhance Parkinson’s disease risk. In particular, our findings reveal that alpha-synuclein overexpression results in a more pronounced loss of nigral dopaminergic neurons in *Gba1*-deficient compared to wild-type mice.

We observed a significant decrease of GCase activity in the midbrain, cortex and striatum of *L444P/+* and *KO/+* mice, with the decrease more prominent in *KO/+* mice. This is perhaps not surprising since *Gba1 KO/+* mice carry a heterozygous knock-out mutation that results in production of a null allele, while *L444P/+* mice carry a heterozygous knock-in mutation that leads to generation of a misfolded mutant protein, which can, at least to some extent, potentially be refolded by chaperones such as heat shock protein 70 (Hsp70) or binding immunoglobulin protein (BiP) in the endoplasmic reticulum (Houck *et al.*, 2012). However, no significant changes in β-hexosaminidase activity were observed in the midbrain, cortex or striatum of *L444P/+* and *KO/+* mice, suggesting that GCase deficiency had no effect on lysosomal content. Interestingly, in humans, differential expression of GCase activity was observed in different brain regions in physiological conditions, with GCase activity being the lowest in the substantia nigra, and the highest in the putamen and amygdala (Gegg *et al.*, 2012). Similarly to humans, GCase activity in different regions of mouse brains in physiological conditions showed differential expression. However, in contrast with the human data, GCase activity was the lowest in the striatum.

We next examined the relationship between GCase activity and alpha-synuclein by analysing alpha-synuclein immunohistochemical staining and protein levels in these models. Prominent accumulation of alpha-synuclein was observed by immunohistochemistry in the olfactory bulb, hippocampus, cortex, striatum and substantia nigra in both *L444P/+* and *KO/+* mice. These findings are consistent with and extend previous observations in other models and in Parkinson’s disease brain that reduced GCase activity results in increased alpha-synuclein levels (Enquist *et al.*, 2007; Manning-Boĝ *et al.*, 2009; Cullen *et al.*, 2011; Mazzulli *et al.*, 2011; Sidransky and Lopez, 2012; Sardi *et al.*, 2011, 2013; Xu *et al.*, 2011, 2014; Cleeter *et al.*, 2013; Osellame *et al.*, 2013; Fishbein *et al.*, 2014). The increase in alpha-synuclein accumulation seems to be more prominent in *KO/+* mice, i.e. in mice displaying greater reduction in GCase activity, suggesting that alpha-synuclein accumulation might perhaps be dependent on GCase activity in a dosage-sensitive manner. We then looked for the presence of insoluble forms of alpha-synuclein in our mouse models by analysing proteinase K resistance, which is an established hallmark of aggregated and insoluble forms of alpha-synuclein (Takeda *et al.*, 1998). Alpha-synuclein aggregates were detected in the hippocampus and striatum of *KO/+* mice. The punctate distribution of proteinase K-resistant alpha-synuclein in *KO/+* mice, similar to the distribution of the physiological protein, suggests synaptic localization of these small aggregates, consistent with the hypothesis that synapses are the initial sites of pathology in synucleinopathies (Calo *et al.*, 2016). Presynaptic alpha-synuclein microaggregates were also previously detected with the use of proteinase K in mouse models of synucleinopathy and in human Parkinson’s disease brains (Beach *et al.*, 2008; Spinelli *et al.*, 2014). We therefore hypothesize that the presence of small, proteinase K-resistant alpha-synuclein species in *KO/+* mice represents early regional synucleinopathy limited to synaptic compartment. Surprisingly, the proteinase K-resistant forms were absent in *L444P/+* mice, suggesting that perhaps alpha-synuclein accumulation present in this mouse model has not yet reached a threshold required for alpha-synuclein aggregation.

Phosphorylation of alpha-synuclein has been suggested to play a key role in alpha-synuclein aggregation and formation of Lewy bodies and neurites. Accumulation of alpha-synuclein phosphorylated at serine 129 (S129) has been shown in the brains of patients with Parkinson's disease, where the phosphorylated protein consists of up to 90% of total alpha-synuclein within Lewy bodies (Sato *et al.*, 2013; Oueslati, 2016). Also, accumulation of S129 phosphorylated alpha-synuclein has been detected in the brain of animal models of synucleinopathies (Migdalska-Richards *et al.*, 2016; Oueslati, 2016). Based on these results, we analysed whether the increase in alpha-synuclein levels was accompanied by a corresponding increase in phosphorylation in both our *L444P/+* and *KO/+* mice, but we did not observe any significant changes in the striatum, midbrain or brainstem. This suggests that our mice show a pre-Parkinson phenotype, where alpha-synuclein starts to accumulate, but pathogenic species of alpha-synuclein (such as S129 phosphorylation) have not yet occurred.

We found no changes in dopaminergic neuron number and no signs of astrogliosis or microgliosis in *KO/+* and *L444P/+* mice, except for increased number of microglial cells in the olfactory bulbs of *L444P/+* mice. These results suggest that, in mice, up to 50% reduction in GCase activity is not sufficient to induce nigral dopaminergic neuron loss or microglia- and astrocyte-mediated inflammation. Post-mortem analysis of human Parkinson-*GBA1* brains showed a prominent loss of dopaminergic neurons in the substantia nigra together with extensive neuroinflammation (Wang *et al.*, 2015). This difference with our results may be due to different factors including species, duration of ‘disease’ and age. Parkinson-*GBA1* brains might also undergo additional genetic or epigenetic changes to produce Parkinson’s disease.

We also found no significant changes in lysosomal function (analysed by cathepsin D and LAMP1 protein levels), autophagy (examined by p62 and LC3 protein levels) or endoplasmic reticulum stress (evaluated by BiP protein levels) in the striatum, midbrain or brainstem of either *L444P/+* or *KO/+* mice. These results suggest that lysosomal function, autophagy and endoplasmic reticulum stress either (i) are not modified by GCase deficiency; (ii) require more than 50% reduction in GCase activity to develop dysfunction; or (iii) require an additional factor (such as loss of dopaminergic neurons or much more pronounced alpha-synuclein accumulation) acting in concert with GCase deficiency to show impairment.

In addition, we looked whether GCase deficiency in *L444P/+* and *KO/+* mice led to the development of behavioural changes related to their olfaction, coordination or cognition, but no significant change was found. This suggests that, in addition to GCase deficiency and alpha-synuclein accumulation, other hallmarks of Parkinson’s disease, such as nigral dopaminergic neuron loss, need to develop in order to produce overt behavioural impairment.

Next, we assessed the effects of AAV-induced overexpression of human alpha-synuclein on the survival of nigrostriatal dopaminergic neurons in the absence or presence of *Gba1* deficiency. The aim of these experiments was to determine the extent to which a decrease in GCase activity may enhance vulnerability to alpha-synuclein pathology. *L444P/+* mice display a relatively less pronounced reduction of GCase activity as compared to *KO/+* mice and, for this reason, were chosen as a better model to investigate potential synergistic effects between *Gba1* deficiency and alpha-synuclein overexpression. The number of dopaminergic neurons was compared in the AAV-injected versus unlesioned substantia nigra in *L444P/+* mice and their wild-type control littermates. Interestingly, we observed (i) no change in neuronal counts in the unlesioned substantia nigra of wild-type control versus *L444P/+* mice; (ii) a slight neuronal loss caused by human alpha-synuclein overexpression in the substantia nigra of wild-type control mice; and (iii) a significantly greater neurodegenerative effect in the AAV-injected substantia nigra of *L444P/+* animals. Taken together, these findings indicate that GCase deficiency is not itself sufficient to induce nigral cell loss but it significantly enhances the toxic effects of alpha-synuclein overexpression. It has been shown previously, in neuronal cultures derived from transgenic mice expressing murine *Gba1* and human alpha-synuclein that the knock-in L444P mutation in *Gba1* gene significantly decreases the rate of human alpha-synuclein degradation, which, as a consequence, leads to an increase in alpha-synuclein steady state levels (Fishbein *et al.*, 2014). Indeed, we found increased alpha-synuclein accumulation in L444P *Gba1* transgenic mice. Hence, we can speculate that the L444P mutation prolongs the half-life of endogenous alpha-synuclein and also of AAV-delivered human alpha-synuclein, which in turn results in total higher alpha-synuclein levels compared to AAV-treated control mice. The extra accumulation of alpha-synuclein in our injected *L444P/+* mice is then sufficient for substantial increase in dopaminergic neuron loss to occur. Our finding may well be relevant to the penetrance of *GBA1* mutations in causing Parkinson’s disease, as it is currently estimated that 10–30% of individuals with *GBA1* mutations will develop Parkinson’s disease by the age of 80 (Anheim *et al.*, 2012; Rana *et al.*, 2013; Alcalay *et al.*, 2014). Our results here suggest that *GBA1* mutations alone are not sufficient to cause Parkinson’s disease, but also require an additional factor such as increased expression of alpha-synuclein (via, for example, epigenetic modifications). Although we cannot exclude that due to the long duration of Parkinson-*GBA1* in human subjects the cumulative effects of the small increase of endogenous alpha-synuclein can reach a threshold value at which pathological changes are triggered.

One of the mechanisms that may contribute to a more severe neurodegeneration in *L444P/+* mice overexpressing alpha-synuclein may be an increased accumulation of pathological, aggregated forms of the protein. This mechanism would be compatible with the hypothesis discussed above since the L444P mutation could prolong alpha-synuclein half-life, enhance its intraneuronal levels and, by doing so, promote alpha-synuclein assembly. Midbrain tissue from AAV-injected wild-type and *L444P/+* mice was immunostained for phosphorylated (at S129) alpha-synuclein as a marker of pathological alpha-synuclein accumulation/aggregation. Protein overexpression caused a dramatic build-up of phosphorylated alpha-synuclein in both control and mutant animals. It is noteworthy, however, that our results revealed a lower density of neurons immunoreactive for the phosphorylated protein in the substantia nigra pars compacta of *L444P/+* animals. This decreased cell density most likely reflects neuronal death, suggesting that neurodegeneration triggered by *Gba1* deficiency may target cells that accumulate phosphorylated (likely aggregated) alpha-synuclein.

We finally examined whether the 30% dopaminergic neuron loss (observed 2 months after AAV-injection) led to a decrease in striatal dopamine and DOPAC content in *L444P/+* mice, but found no evidence for any changes. These data are consistent with earlier observations showing that intranigral injections of human-alpha-synuclein AAVs do not necessarily deplete striatal dopamine concentrations, even in the presence of overt degeneration of nigral dopaminergic neurons (Decressac *et al.*, 2012; Lastres-Becker *et al.*, 2012). This may be due to the fact that, when AAVs are injected directly into the ventral mesencephalon, the ensuing toxicity preferentially targets neuronal cell bodies in the substantia nigra; under these experimental conditions, compensatory mechanisms may help preserve dopaminergic function at the level of striatal terminals.

**Figure 7:**
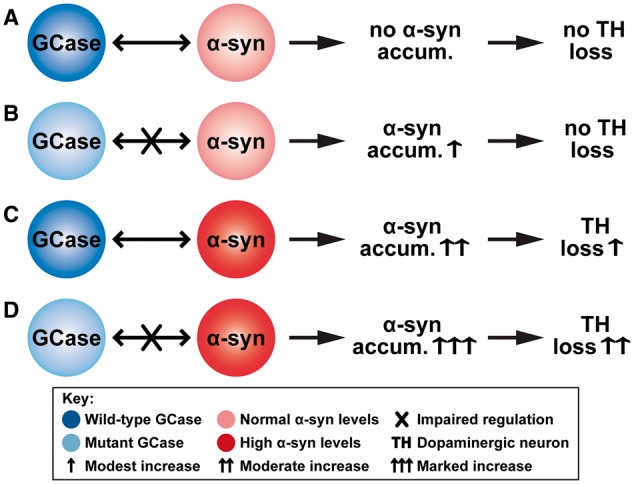
**Potential mechanism linking GCase, alpha-synuclein and dopaminergic neurons.** We assume that GCase regulates the alpha-synuclein levels and that sufficiently high levels of alpha-synuclein lead to dopaminergic neuron loss. (**A**) Wild-type GCase regulates alpha-synuclein levels. Thus, under normal conditions, there is no alpha-synuclein accumulation and so no dopaminergic neuron loss. (**B**) Mutant GCase can only partially regulate alpha-synuclein levels. This leads to modest alpha-synuclein accumulation, which, however, is not high enough to cause any neuron loss. (**C**) High levels of alpha-synuclein are still partially regulated by wild-type GCase. This leads to moderate alpha-synuclein accumulation, resulting in modest neuron loss. (**D**) The high alpha-synuclein levels can no longer be regulated by the mutant GCase. This leads to marked alpha-synuclein accumulation, which, in turn, results in substantial neuron loss.

In conclusion, we have shown that GCase deficiency alone causes prominent increase in alpha-synuclein accumulation in *L444P/+* and *KO/+* mice, but does not lead to the development of other behavioural and biochemical features of Parkinson’s disease during the lifespan of the mouse. Similarly, overexpression of alpha-synuclein in the substantia nigra does not by itself always lead to significant neuron loss. Yet, the combination of GCase deficiency and increased alpha-synuclein levels in our mouse models showed pronounced loss of dopaminergic neurons (Fig. 7). However, we and others have previously demonstrated in a *Drosophila* model that L444P mutation can induce TH loss without concomitant expression of alpha-synuclein (Davis *et al.*, 2016; Sanchez-Martin *et al.*, 2016). The difference in the toxicity of the *GBA1* mutation may reflect species specificity.

The data presented here suggest that Parkinson-*GBA1* is more likely to develop if alpha-synuclein levels are increased either by polymorphism-determined increased transcription, post-translational changes in protein stability, or potentially epigenetic modifications or an environmental influence that affects alpha-synuclein expression/turnover, all factors that can add to the long-term progressive accumulation of alpha-synuclein in *GBA1* patients. This can be further explored by comparison of genetic, epigenetic and environmental factors in aged *GBA1* mutation positive individuals with and without Parkinson’s disease. This study is currently underway in our group.

## Supplementary Material

Supplementary Figure S1

Supplementary Figure S2

Supplementary Figure S3

Supplementary Figure S4

Supplementary Table S1

Supplementary Table S2

Supplementary Table S3

Supplementary Material

## Abbreviation

GCase: glucocerebrosidase
