# Supplementary material for: The L444P Gba1 mutation enhances alpha-synuclein induced loss of nigral dopaminergic neurons in mice
Source: Brain. 2017 Sep 6;140(10):2706–21. doi: 10.1093/brain/awx221 (PMC5841155; doi:10.1093/brain/awx221)
Supplement: Supplementary Figure S1 [file awx221_supp_figure1.pdf]

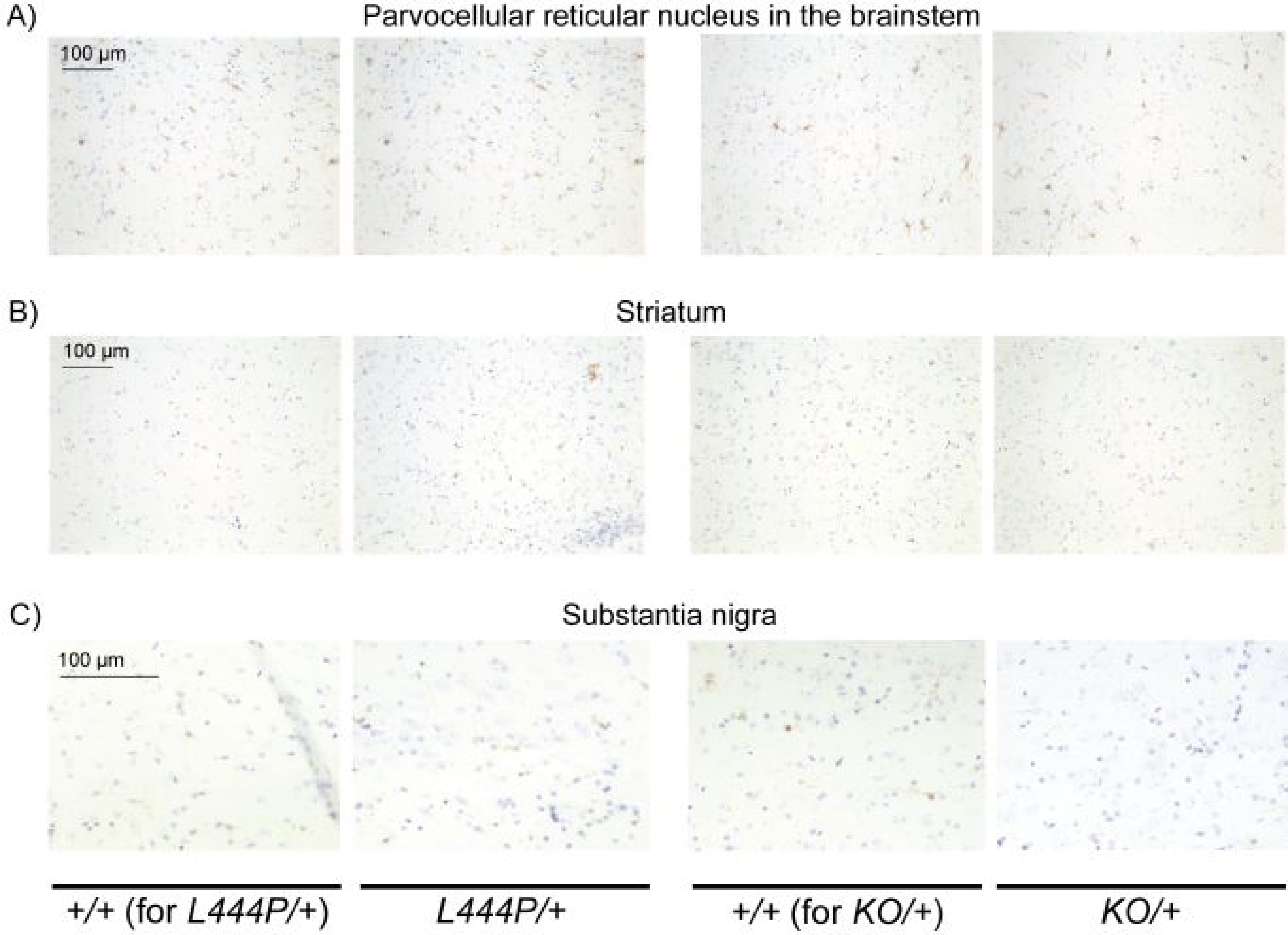

A)
Parvocellular reticular nucleus in the brainstem
100 µm
B)
Striatum
100 µm
C)
Substantia nigra
100 µm
+/+ (for L444P/+)
L444P/+
+/+ (for KO/+)
KO/+
