# Supplementary figures and images for: The L444P Gba1 mutation enhances alpha-synuclein induced loss of nigral dopaminergic neurons in mice
Source: Brain. 2017 Sep 6;140(10):2706–21. doi: 10.1093/brain/awx221 (PMC5841155; doi:10.1093/brain/awx221)

# Striatum

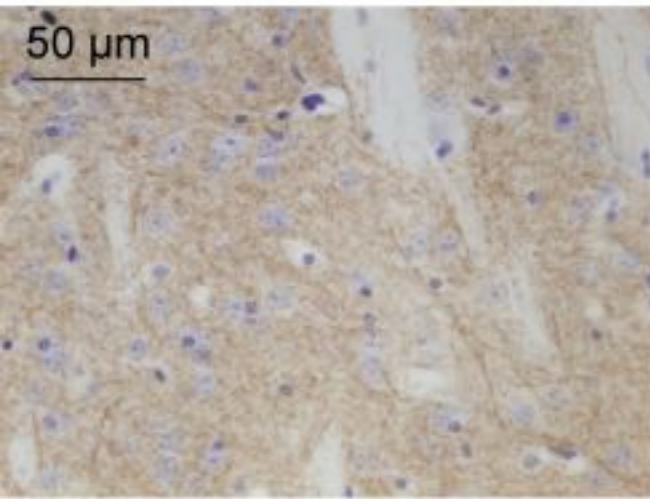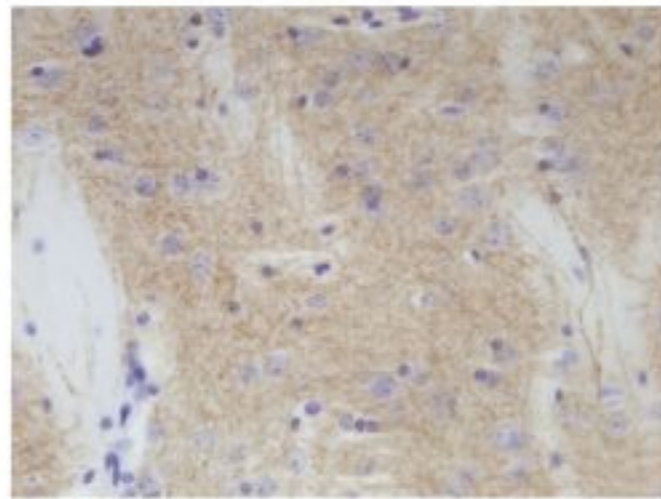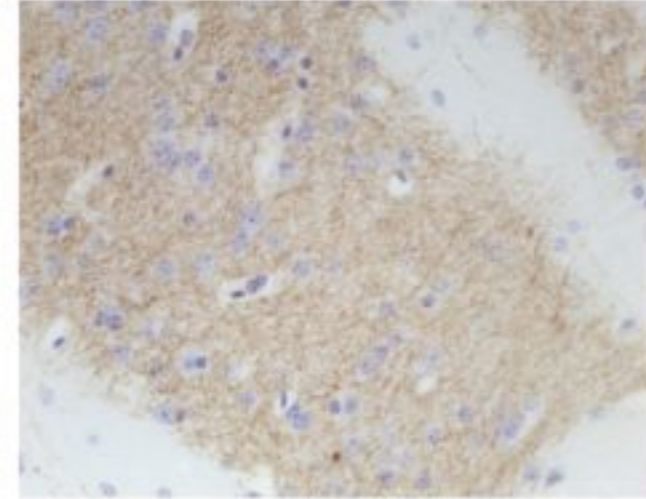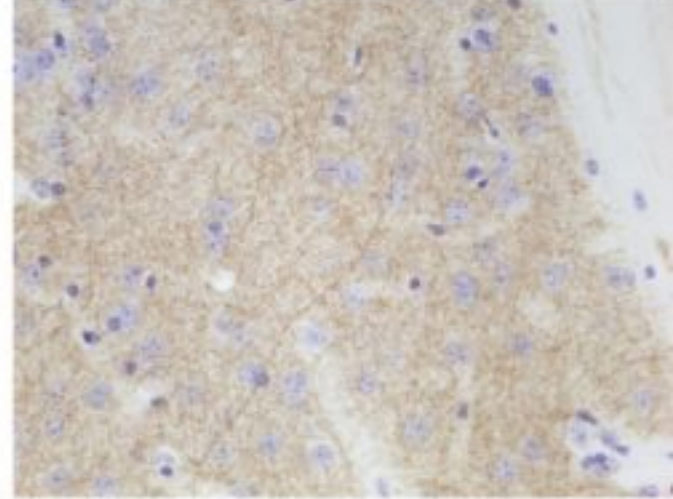

*+/+* (for *L444P/+*)

*L444P/+*

*+/+* (for *KO/+*)

*KO/+*

Supplement: Supplementary Figure S3 [file awx221_supp_figure3.pdf]

# Granule cell layer of the olfactory bulb

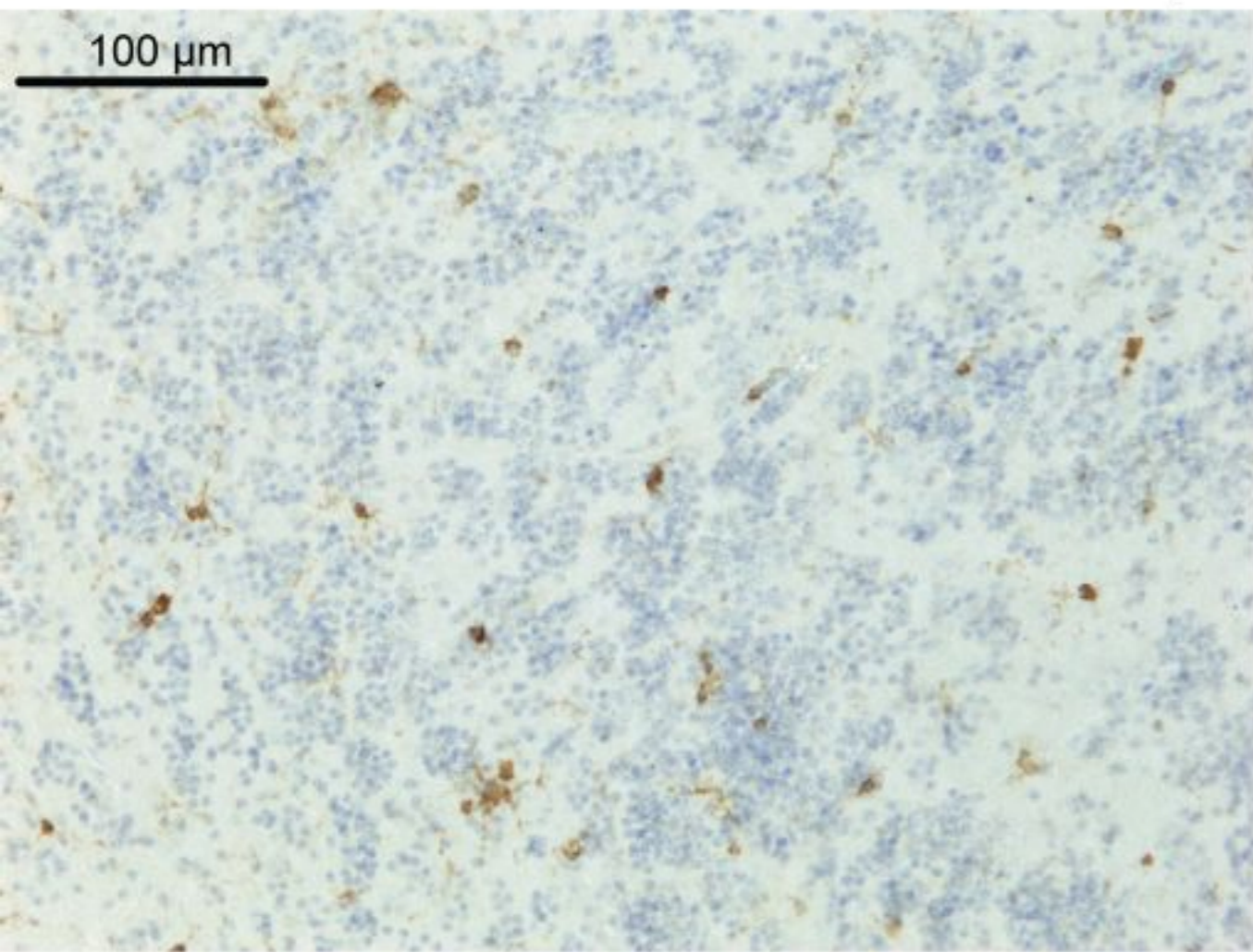

+/+ (for *L444P*/+)

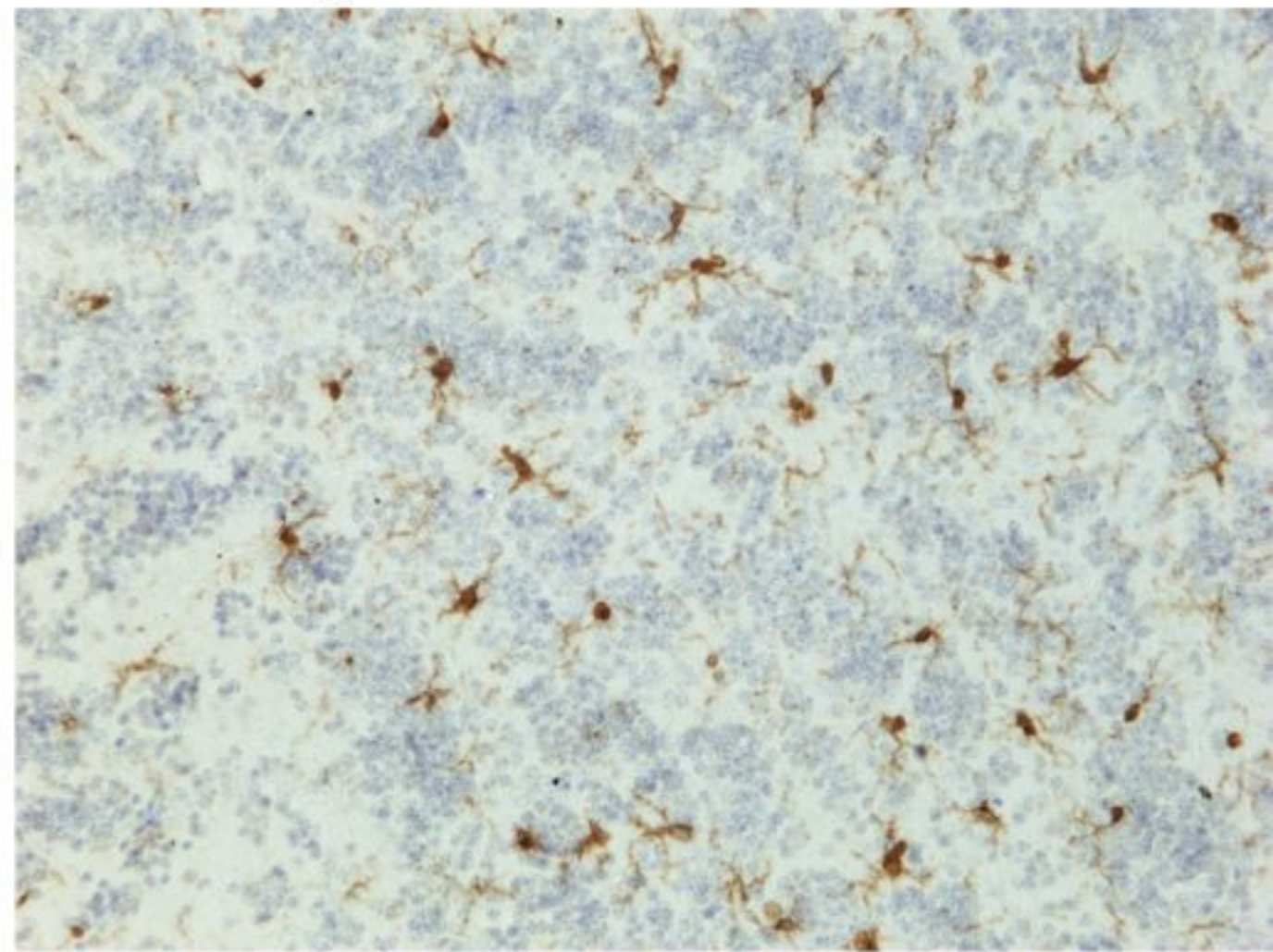

*L444P*/+

Supplement: Supplementary Figure S4 [file awx221_supp_figure4.pdf]
