# Supplementary material for: The L444P Gba1 mutation enhances alpha-synuclein induced loss of nigral dopaminergic neurons in mice
Source: Brain. 2017 Sep 6;140(10):2706–21. doi: 10.1093/brain/awx221 (PMC5841155; doi:10.1093/brain/awx221)
Supplement: Supplementary Table S1 [file awx221_supp_table1.pdf]

Supplementary Table 1.

Brain region \ Protein	Cathepsin D	p62	Bip	GFAP	Lamp1	LC3B
Brainstem	-7%	+6%	+9%	-15%	-13%	-1%
Midbrain	-9%	+7%	+13%	-10%	-7%	-10%
Striatum	-5%	+5%	+8%	-16%	-5%	+13%

Legend: - decrease in protein levels in *L444P/+* mice compared to +/+ control littermates
+ increase in protein levels in *L444P/+* mice compared to +/+ control littermates
