# Supplementary material for: The L444P Gba1 mutation enhances alpha-synuclein induced loss of nigral dopaminergic neurons in mice
Source: Brain. 2017 Sep 6;140(10):2706–21. doi: 10.1093/brain/awx221 (PMC5841155; doi:10.1093/brain/awx221)
Supplement: Supplementary Table S2 [file awx221_supp_table2.pdf]

Supplementary Table 2.

Brain region \ Protein	Cathepsin D	p62	GFAP	Lamp1	LC3B
Brainstem	-2%	+9%	-11%	-14%	+9%
Midbrain	-5%	+13%	-2%	-10%	+6%
Striatum	-10%	+4%	-5%	-2%	+3%

Legend: - decrease in protein levels in *L444P/+* mice compared to +/+ control littermates
+ increase in protein levels in *L444P/+* mice compared to +/+ control littermates
