# Supplementary material for: The L444P Gba1 mutation enhances alpha-synuclein induced loss of nigral dopaminergic neurons in mice
Source: Brain. 2017 Sep 6;140(10):2706–21. doi: 10.1093/brain/awx221 (PMC5841155; doi:10.1093/brain/awx221)
Supplement: Supplementary Table S3 [file awx221_supp_table3.pdf]

Supplementary Table 3.

L444P/+	+/+<br>(for *L444P/+*)	*KO/+*	+/+<br>(for *KO/+*)
Buried pellet test<br>(the latency to find and start eating the piece of chocolate)			
141s	135s	81s	70s
Pole test – t-turn<br>(time to orient downward)			
1.25s	1.3s	2.28s	1.42s
Pole test – t-total<br>(total time to descend)			
4.67s	4.5s	5.42s	5.32
Novel object recognition test<br>(time spent exploring novel object divided by the sum of time spent exploring both novel and familiar object)			
0.47	0.51	0.57	0.53
