# Supplementary material for: The L444P Gba1 mutation enhances alpha-synuclein induced loss of nigral dopaminergic neurons in mice
Source: Brain. 2017 Sep 6;140(10):2706–21. doi: 10.1093/brain/awx221 (PMC5841155; doi:10.1093/brain/awx221)
Supplement: Supplementary Material [file awx221_supp_material.pdf]

Supplementary Material

Primary antibodies

Alpha-synuclein (Abcam ab1903, 1:2000 or Cell Signaling D37A6, 1:1000), phospho S129 alpha-synuclein (Abcam ab51253, 1:2000), SQSTM1/p62 (Abcam ab56416, 1:2000), Cathepsin D (Abcam ab6313, 1:2000), GRP78 BiP (Abcam ab21685, 1:2000), LC3B (Abcam ab48394, 1:2000), LAMP1 (Abcam ab24170, 1:500), Glial Fibrillary Acidic Protein Antibody, clone GA5 (Millipore MAB3402, 1:1000) and β-actin (Abcam ab6276, 1:10000).

Behavioral tests

All mice were pre-handled for 1 to 2 minutes every day for four days prior to testing. In each behavioral test eight *L444P/+* and ten +/+ control littermates, and nine *KO/+* and nine +/+ control littermates were used. All the tests were performed at least twice.

Buried pellet test. Olfactory function was analyzed with the buried pellet test using a clean cage (50 cm $L \times 25$ cm $W \times 20$ cm H) containing a removable partition with the floor of the cage covered with 3 cm fresh deep bedding material. For 3 consecutive days before the test, a piece of chocolate was placed in a home cage of each tested mouse to habituate it to the new food. Prior to the test, mice were deprived of food overnight, but left with free water access. On the day of the test, each mouse was placed in a fresh cage. After 5 minutes of habituation time, the piece of chocolate was placed underneath the bedding (behind the partition), the partition was removed, and the latency to find and start eating the piece of chocolate (up to 5 minutes) was measured using a stopwatch.

Pole test. Motor function was investigated with the pole test using a vertical 60 cm long wooden pole (3 cm in diameter) that was placed in the mouse home cage. The entire procedure spanned over 5 days and involved two stages: training and testing. On days 1 to 4, during the training sessions, mice were placed head-up on top of the pole, left to orient themselves downward and descend the length of the pole back into their home cage. Each mouse was subjected to five trials per each session. On the testing session, mice were given three trials, and time to orient downward (t-turn) and total time to descend (t-total) were measured using two stopwatches. The best performance over the three trials was used for both transgenic and wild-type mice.

Novel object recognition test. Cognitive function was analyzed with the novel object recognition test using a plexiglass box (50 cm $L \times$ 25 cm $W \times$ 20 cm H). The entire procedure spanned over 5 days and involved three stages: habituation, training and testing. On days 1 to 3, animals were brought to the testing room 15 minutes before the experiment, and then individually habituated to the box in the absence of objects for 15 minutes. On day 4, each mouse was subjected to a training session. During the training session, two identical objects were placed at two opposite corners of the box. Each mouse was individually allowed to explore the identical objects for 10 minutes and then was placed back in its home cages. 24 hours later, mice were subjected to a testing session. During 5 minutes testing session, each mouse was individually placed back to the same box, where the training session occurred, but one of the two familiar objects was switched to a novel one. All objects used in this study differed in shapes and colors, but were very similar in size. The objects were fixed to the corners of the box to avoid their accidental movement. The box and objects were always thoroughly cleaned with 70%

ethanol after each mouse to exclude the existence of olfactory prompts. All trials were recorded with a video camera and the videos were subsequently scored blind of genotype using a stopwatch. Object exploration time was defined as the length of time that a mouse spent investigating, by a direct oronasal contact or close approach (less than 1 cm), sniffing or pawing the object. Sitting or standing on the object was not recognized as the exploration. In the training session, the location preference was calculated as time spent exploring one of the identical objects divided by the sum of time spent exploring the pair of identical object. In the test session, the recognition index was calculated as time spent exploring novel object divided by the sum of time spent exploring both novel and familiar object. Location preference was used as an environmental control, which should be 1/2, to rule out the influence of the location of the object. Animals with a total exploration time of less than 5 seconds during the testing session were excluded. If the mouse during the testing session spent longer investigating a novel object than the familiar one, this was taken as evidence for recognition.

Supplementary Figures and Tables Legend

Supplementary Figure 1. Ionized calcium binding adaptor molecule 1 (Iba1) immunohistochemistry in *L444P/+* and *KO/+* mouse brains. A-C) No changes in the Iba1 staining were found in the parvocellular reticular nucleus of the brainstem (A), striatum (B) and substantia nigra (C) of *KO/+* and *L444P/+* mice compared to their corresponding +/+ control littermates. Scale bars = 100 μm. Representative images are shown. Three mice of each genotype were analyzed.

Supplementary Figure 2. Glial fibrillary acidic protein (GFAP) immunohistochemistry in *L444P/+* and *KO/+* mouse brains. A-C) No changes in the GFAP staining were found in the parvocellular reticular nucleus of the brainstem (A), striatum (B) and substantia nigra (C) of *KO/+* and *L444P/+* mice compared to their corresponding +/+ control littermates. Scale bars = 100 μm. Representative images are shown. Three mice of each genotype were analyzed.

Supplementary Figure 3. Tyrosine hydroxylase (TH) immunohistochemistry in the striatum of *L444P/+* and *KO/+* mice. No changes in the TH staining were found in the striatum of *KO/+* and *L444P/+* mice compared to their corresponding +/+ control littermates. Scale bars = 50 μm. Representative images are shown. Three mice of each genotype were analyzed.

Supplementary Figure 4. Ionized calcium binding adaptor molecule 1 (Iba1) immunohistochemistry in the granule cell layer of the olfactory bulb of *L444P/+* mice. Increased Iba1 staining was found in the granule cell layer of the olfactory bulb of *L444P/+* mice compared to their corresponding +/+ control littermates. Scale bars = 100 μm. Representative images are shown. Three mice of each genotype were analyzed.

Supplementary Table 1. Changes in cathepsin D, p62, Bip, GFAP, Lamp1 and LC3B protein levels in *L444P/+* mice.

Supplementary Table 2. Changes in cathepsin D, p62, GFAP, Lamp1 and LC3B protein levels in *KO/+* mice.

Supplementary Table 3. Summary of behavioral tests results for *L444P/+* mice and *KO/+* mice.
